# Biorecovery of cobalt and nickel using biomass-free culture supernatants from *Aspergillus niger*

**DOI:** 10.1007/s00253-019-10241-2

**Published:** 2019-11-28

**Authors:** Yuyi Yang, Wenjuan Song, John Ferrier, Feixue Liu, Laszlo Csetenyi, Geoffrey Michael Gadd

**Affiliations:** 1 Geomicrobiology Group, School of Life Sciences, University of Dundee, Scotland, DD1 5EH UK; 2 Xinjiang Institute of Ecology and Geography, Chinese Academy of Sciences, Urumqi, 830011 China; 3 Concrete Technology Group, Department of Civil Engineering, University of Dundee, Scotland, DD1 5EH UK

**Keywords:** *Aspergillus niger*, Biorecovery, Extracellular polymeric substances, Fluorescence quenching, Cobalt, Nickel, Oxalate

## Abstract

In this research, the capabilities of culture supernatants generated by the oxalate-producing fungus *Aspergillus niger* for the bioprecipitation and biorecovery of cobalt and nickel were investigated, as was the influence of extracellular polymeric substances (EPS) on these processes. The removal of cobalt from solution was >90% for all tested Co concentrations: maximal nickel recovery was >80%. Energy-dispersive X-ray analysis (EDXA) and X-ray diffraction (XRD) confirmed the formation of cobalt and nickel oxalate. In a mixture of cobalt and nickel, cobalt oxalate appeared to predominate precipitation and was dependent on the mixture ratios of the two metals. The presence of EPS together with oxalate in solution decreased the recovery of nickel but did not influence the recovery of cobalt. Concentrations of extracellular protein showed a significant decrease after precipitation while no significant difference was found for extracellular polysaccharide concentrations before and after oxalate precipitation. These results showed that extracellular protein rather than extracellular polysaccharide played a more important role in influencing the biorecovery of metal oxalates from solution. Excitation–emission matrix (EEM) fluorescence spectroscopy showed that aromatic protein-like and hydrophobic acid-like substances from the EPS complexed with cobalt but did not for nickel. The humic acid-like substances from the EPS showed a higher affinity for cobalt than for nickel.

## Introduction

Microorganisms can play an important role in both the remediation and biorecovery of metals (Gadd 2010; Liang and Gadd 2017). Although metals cannot be degraded into harmless compounds, their chemical form, mobility, toxicity, and bioavailability can be changed via the growth, metabolism, and metabolic products of microorganisms (Peng et al. 2018). Metal immobilization or recovery from solution can be achieved by bioprecipitation where metals are transformed from soluble species to insoluble compounds, such as oxides, carbonates, phosphates, oxalates, and sulfides (Haferburg and Kothe 2007; Tsezos 2009; Gadd et al. 2014; Liang and Gadd 2017). For example, Fe, Zn, and Cd in wastewater showed more than 99% precipitation rates in downflow fluidized bed reactors containing sulfate-reducing bacteria (SRB) (Gallegos-Garcia et al. 2009). Biological sulfide precipitation combined with solvent extraction can even result in nanosized metal sulfides for biorecovery (Nanusha et al. 2019). Microbially induced calcite precipitation (MICP) is also a promising biotechnology for recovery or immobilization of metals from wastewater or groundwater (Li et al. 2014; Li and Gadd 2017a, b; Kumari et al. 2016; Zhu and Dittrich 2016; Torres-Aravena et al. 2018). A total of 61% of calcium and 56% of strontium precipitation rates were obtained in porous media reactors via MICP mediated by the bacterium *Sporosarcina pasteurii* (Lauchnor et al. 2013). Calcium recovery of more than 90% was achieved from a calcium-rich industrial wastewater using bacterial MICP (Hammes et al. 2003). Supernatants obtained from ureolytic fungi are also very efficient in forming copper and other metal carbonate nanoparticles for biorecovery (Li et al. 2014, 2015, 2019; Li and Gadd, 2017a, b; Liu et al. 2019).

Organic acids, e.g. oxalic acid produced by fungi, can also play an important role in the immobilization or biorecovery of metals (Clarholm et al. 2015; Gadd 1999; Gadd et al. 2014; Mishra et al. 2017; Yang et al. 2019). Wood-rotting fungi can immobilize toxic metals in a metal-amended substrate by precipitation as metal oxalates (Kaewdoung et al. 2016). Lead immobilization via oxalates was also found with fluorapatite and *Aspergillus niger* (Li et al. 2016). In previous studies, it was also found that metabolites of the geoactive fungi *Aspergillus niger* and *Beauveria caledonica* could immobilize rare earth elements and toxic metals as oxalates (Fomina et al. 2005; Kang et al. 2019). Chemical oxalate precipitation is also widely used for the recovery of actinides (Abraham et al. 2014). High purity magnesium oxalate was obtained from Uyuni salar brine via chemical oxalate precipitation (Tran et al. 2013). Nickel is a primary co-existing element in Co minerals (Hazen et al. 2017), while industrially, cobalt, and nickel are also normal elements used in several kinds of batteries (Lupi et al. 2005; Rodrigues and Mansur 2010; Chen et al. 2011). In this research, we have used fungal products for biorecovery of cobalt and nickel from solution to (1) investigate the efficiency of culture supernatants from *Aspergillus niger* for cobalt and nickel biorecovery, (2) identify the bioprecipitation products formed, (3) determine the possible influence of extracellular polymeric substances (EPS) on the biorecovery process. This study will provide insights into the roles of fungal metabolites in metal–mineral interactions and their potential for metal biorecovery from solution.

## Methods and materials

### Microorganism and media

The experimental fungus used in this study was *Aspergillus niger* (ATCC 1015), which was incubated on malt extract agar slants (Lab M Limited, Heywood, Lancashire, UK) at 25 °C in the dark for 7 days to prepare spore suspensions for inoculation of liquid media according to a previous study (Kang et al. 2019). Modified Czapek-Dox (MCD) medium consisted of (g L^−1^ Milli-Q water): d-glucose, 30; NaNO_3_, 3; Na_2_HPO_4_, 1; MgSO_4_·7H_2_O, 0.5; KCl, 0.5; and FeSO_4_·7H_2_O, 0.01. All components were individually prepared as 100 mL stock solutions at the appropriate concentration and sterilized at 115 °C for 20 min prior to experiments. The initial pH of MCD media was adjusted to pH 5.5 using 1 M HCl before autoclaving. The initial spore concentration in the medium was 5 × 10^5^ mL^−1^. *A. niger* was grown in 100-mL liquid medium in 250-mL Erlenmeyer flasks incubated on a shaker at 150 rpm at 25 °C in the dark. Biomass-free culture supernatants after 1- or 2-week incubation were obtained by filtering the medium through 0.45-μm cellulose nitrate membrane filters (Minisart syringe filters, Sartorius, Göttingen, Germany). All chemicals were obtained from Sigma-Aldrich Ltd., St. Louis, MO, USA unless stated otherwise.

### Recovery of cobalt and nickel via bioprecipitation by fungal supernatants

Different concentrations of cobalt, nickel, and their mixture were used to examine their recovery from solution as biogenic minerals. Aliquots from stock solutions of 100 mM CoCl_2_∙4H_2_O and/or NiCl_2_∙6H_2_O were added to 27 mL biomass-free supernatants to reach a total volume of 30 mL, with or without the addition of Milli-Q H_2_O, in 50-mL tubes and mixed at 60 rpm for 24 h on a tube rotator. The resulting precipitate and supernatants from the tubes were collected after centrifugation (2012 g, 30 min). The concentrations of cobalt and nickel in the supernatants were measured by atomic absorption spectrophotometry (AAS) (AAnalyst 400 Atomic Absorption Spectrophotometer, PerkinElmer Ltd., Beaconsfield, UK). All experiments were carried out at least in triplicate.

Environmental scanning electron microscopy (ESEM) and X-ray diffraction (XRD) analysis of the collected precipitates were used to examine the morphology and mineralogical composition of the precipitated minerals. Detailed experimental procedures can be found in previous publications (Li et al. 2014; Li and Gadd 2017a). Precipitates were dried in a desiccator at ambient temperature for at least 5 days, mounted on aluminum stubs using carbon adhesive tape and coated with 10 nm Au/Pd using a Cressington 208HR sputter coater (Cressington, Watford, UK) and examined using a Philips XL30 ESEM (Philips XL 30 ESEM FEG) operating at an accelerating voltage of 15 kV. The mineral phases of the precipitates were identified using a Hiltonbrooks X-ray diffractometer (HiltonBrooks Ltd., Crewe, UK) equipped with a single graphite crystal monochromatic CuKα chronometer (30 mA, 40 kV).

### Influence of extracellular polymeric substances on recovery of cobalt and nickel

Supernatants were collected from MCD media after growth of *A. niger* and then dialyzed using dialysis membrane to obtain an extracellular polymeric substances (EPS) solution. Twenty-five millimolar of sodium oxalate was dissolved in the EPS solution, and the pH was adjusted to pH 2.2 using 1 M HCl, the same value as in oxalate-free supernatants. The EPS solution containing oxalate was then used to precipitate cobalt and nickel from single and mixed solutions and using a 25-mM sodium oxalate solution treatment as a control. The content of extracellular polysaccharide and protein before and after precipitation was determined using the phenol-sulfuric acid method (DuBois et al. 1956) and Bradford method (Bradford 1976), respectively.

### Excitation emission matrix fluorescence spectroscopy and quenching titrations

A Hitachi F-7000 fluorescence spectroscope (Hitachi, Tokyo, Japan), equipped with a 1.0-cm quartz cell and thermostatic bath, was used to determine the excitation emission matrix (EEM) spectra of EPS obtained from the *A. niger* culture supernatants. EEM spectra were obtained according to published methods (Song et al. 2012; Wang et al. 2018). For the quenching titration, the EPS solution was titrated with incremental microliter additions of Co(II) and Ni(II) solution at 308 K (35 °C). After each addition of metal salt, the solution was mixed using a magnetic stirrer for 15 min and EEM spectra were recorded during this process. Fifteen minutes was set as the equilibrium time because the fluorescence intensities showed almost no change after 15-min reaction.

## Results

### Recovery of cobalt and nickel as oxalate minerals using *A. niger* biomass-free culture supernatants

**Fig. 1:**
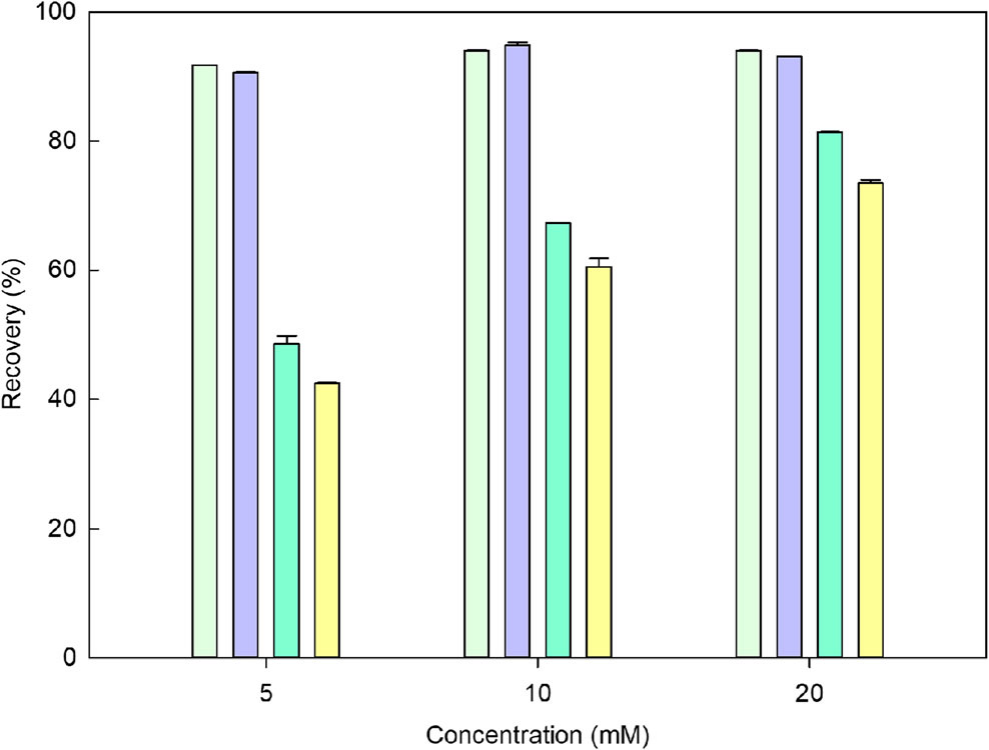
Removal of cobalt and nickel from solution using supernatants of *A. niger* (  7-day supernatants + Co;  14-day supernatants + Co;  7-day supernatants + Ni;  14-day supernatants + Ni). Bars are the standard error of the mean (*n* = 3)

**Fig. 2:**
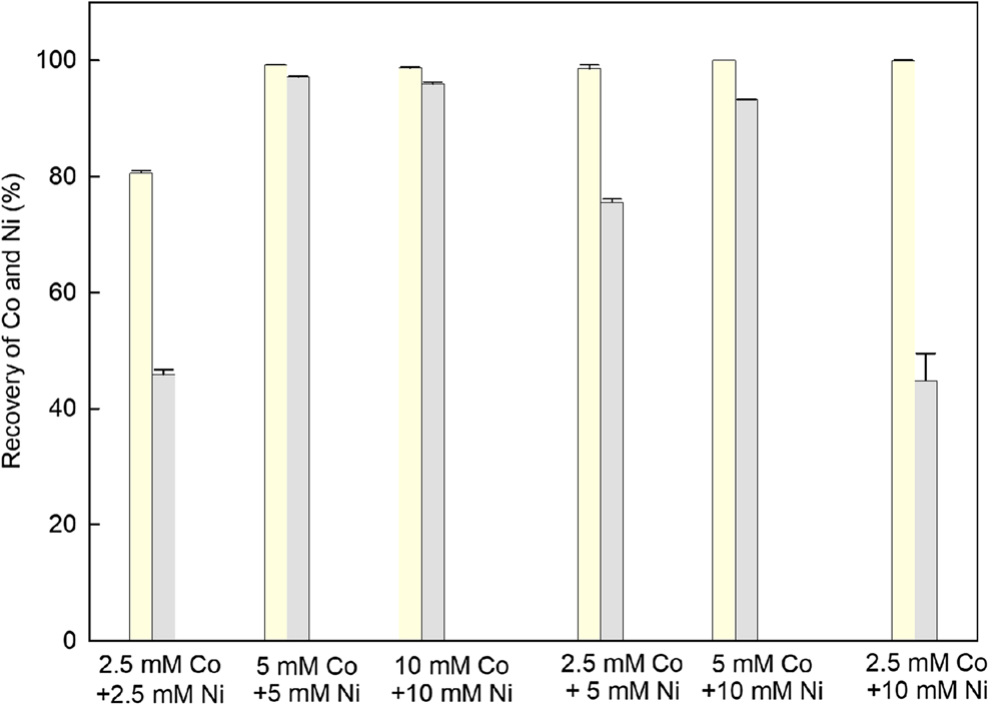
The removal of cobalt and nickel from a mixed solution using supernatants of *A. niger* collected after 7 days (  Co;  Ni). Bars are the standard error of the mean (*n* = 3)

The biomass-free supernatants collected from *A. niger* liquid cultures after 7 and 14 days exhibited excellent precipitation and recovery of cobalt from solution (Fig. 1). Recovery of cobalt from solution was above 90% for all tested cobalt concentrations. There was no significant difference in cobalt recovery between supernatants harvested after 7 and 14 days (*p* > 0.05). Nickel precipitation and recovery from *A. niger* 7- and 14-day-old supernatants increased as the concentration of nickel increased from 5 to 20 mM. Maximum recovery (81.44%) of nickel occurred at a concentration of 20 mM NiCl_2_∙6H_2_O using supernatants collected after 7 days. The 7-day-old supernatants collected from *A. niger* cultures exhibited significantly higher recovery of nickel than supernatants collected after 14 days (*p* < 0.05). Since the 7-day old supernatants exhibited such high recovery rates for both cobalt and nickel, these were used for subsequent experiments. Figure 2 shows the recovery of cobalt and nickel from solutions with different concentration ratios (1:1, 1:2 and 1:4). The results revealed that the removal of cobalt from a mixture of cobalt and nickel were all above 98%, except for 2.5 mM cobalt and nickel. The recovery of nickel from the mixture was in the range 44.9–93.1%. Recovery of cobalt and nickel from the supernatants therefore depended on the mixture ratios of the two metals.

### Influence of extracellular polymeric substances on recovery of cobalt and nickel

**Fig. 3:**
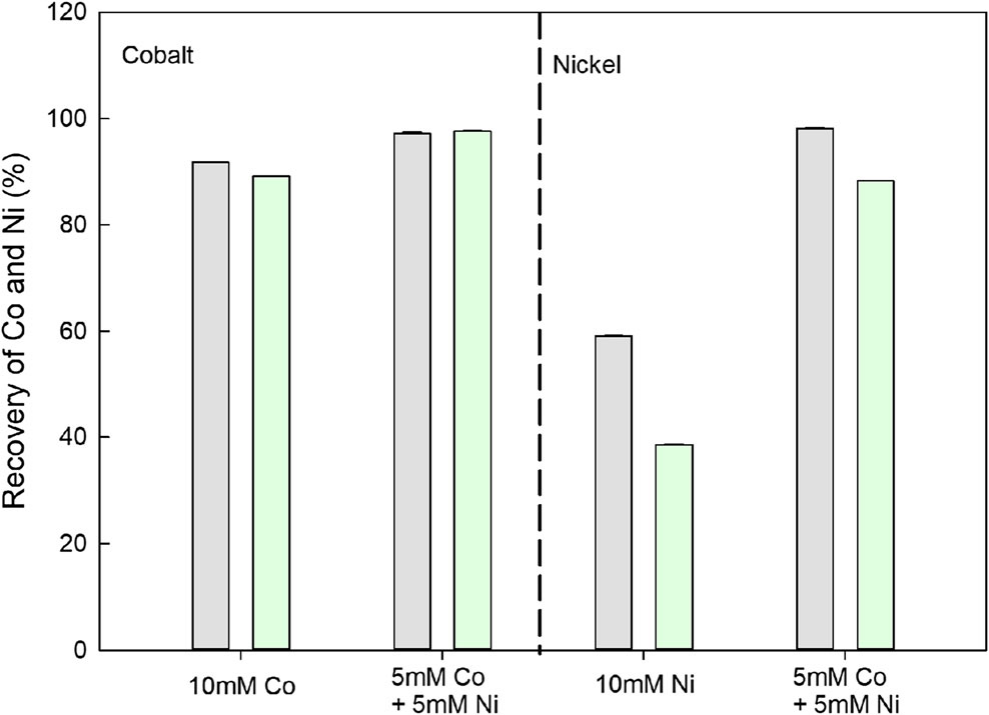
The removal of cobalt and nickel from solutions using oxalate with or without extracellular polymeric substances (EPS) ( oxalate;  oxalate + EPS). Bars are the standard error of the mean (*n* = 3)

**Fig. 4:**
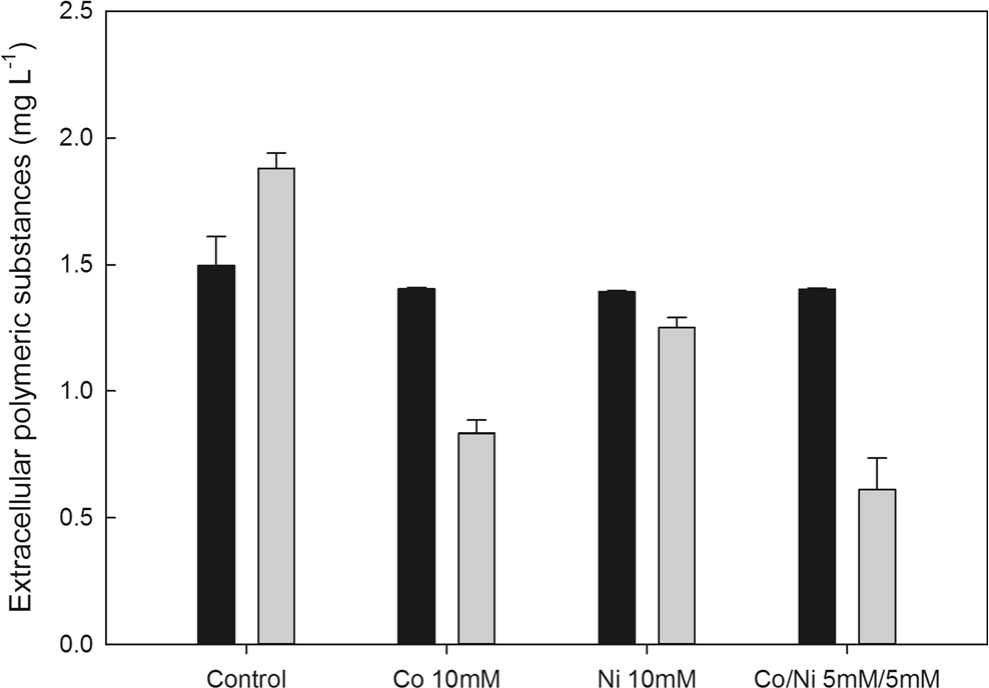
Changes in extracellular polymeric substances (EPS) concentration before and after removal of cobalt and nickel using oxalate with EPS ( extracellular polysaccharide;  extracellular protein). Bars are the standard error of the mean (*n* = 3)

The influence of EPS on the efficiency of metal removal was investigated. From Fig. 3, it can be seen that EPS did not influence the recovery of cobalt from solution. However, the presence of EPS decreased nickel recovery significantly. Nickel recoveries from 10 mM nickel and a mixed solution of 5 mM cobalt and 5 mM nickel using a chemical oxalate solution were 59.1% and 98.2%, respectively. When using an EPS solution containing oxalate, nickel recoveries from 10 mM nickel and a mixed solution of 5 mM cobalt and 5 mM nickel were 38.5% and 88.3%, respectively. The polysaccharide concentration in the EPS solution before precipitation was 1.50 mg L^−1^ (Fig. 4). Extracellular polysaccharide concentrations in solution after precipitation of cobalt and nickel from single solutions and their mixture were 1.40, 1.39, and 1.40 mg L^−1^, respectively. An insignificant decrease in the extracellular polysaccharide concentration therefore occurred after oxalate precipitation. The concentration of extracellular protein before precipitation was 1.88 mg L^−1^. Extracellular protein concentrations in solution after precipitation with cobalt, nickel, and their mixture were 0.83, 1.25, and 0.61 mg L^−1^, respectively. A significant reduction of extracellular protein in solution therefore resulted after oxalate precipitation (*p* < 0.05). It can also be noted that more extracellular protein disappeared during the cobalt treatment compared to the nickel treatment.

### Characterization of cobalt and nickel oxalate minerals

**Fig. 5:**
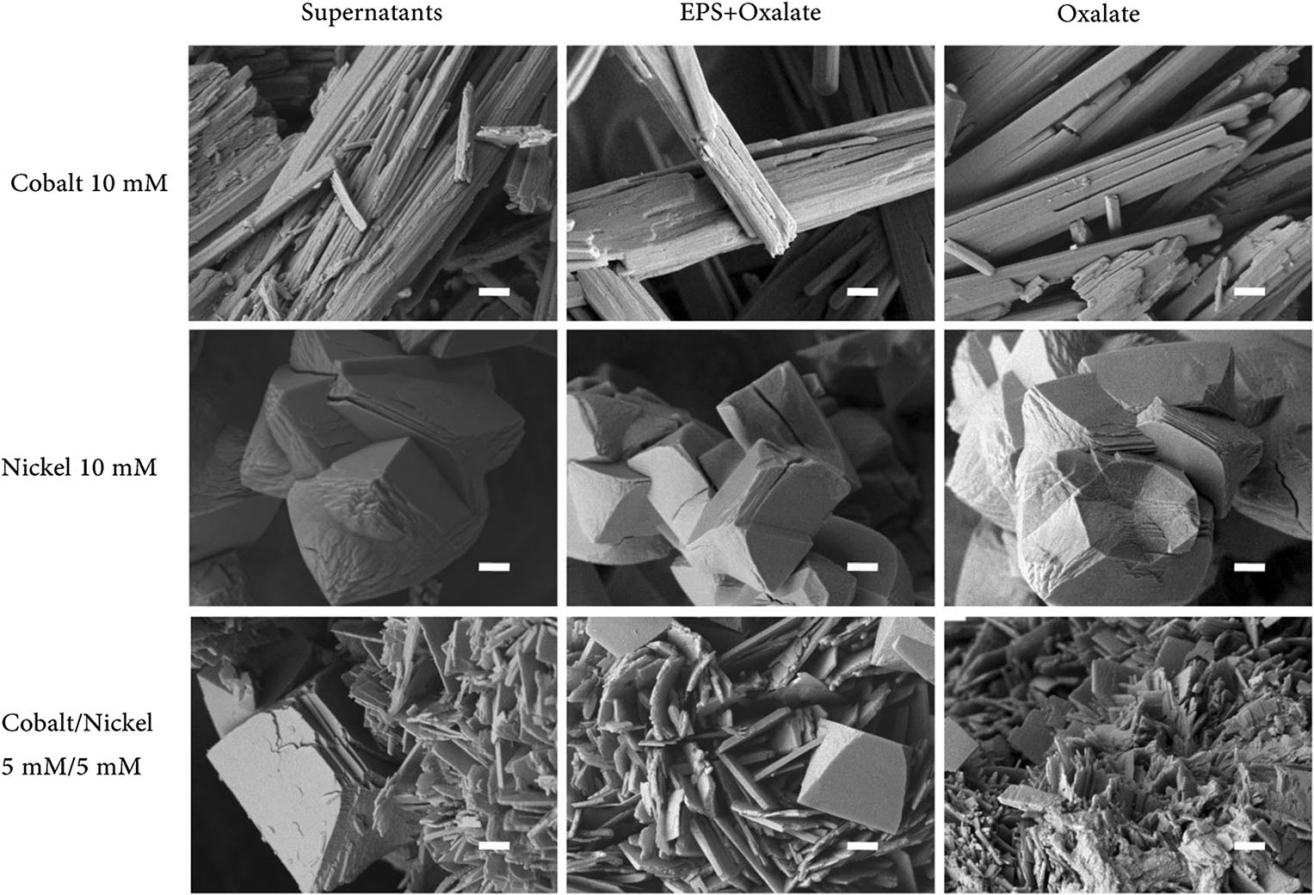
Scanning electron microscopy images of the Co/Ni precipitates (10 mM cobalt, 10 mM nickel and a mixture of 5 mM cobalt and 5 mM nickel reacted with culture supernatants, oxalate with EPS and oxalate, respectively). Scale bars (1 μm). Typical images are shown from several separate examinations

**Fig. 6:**
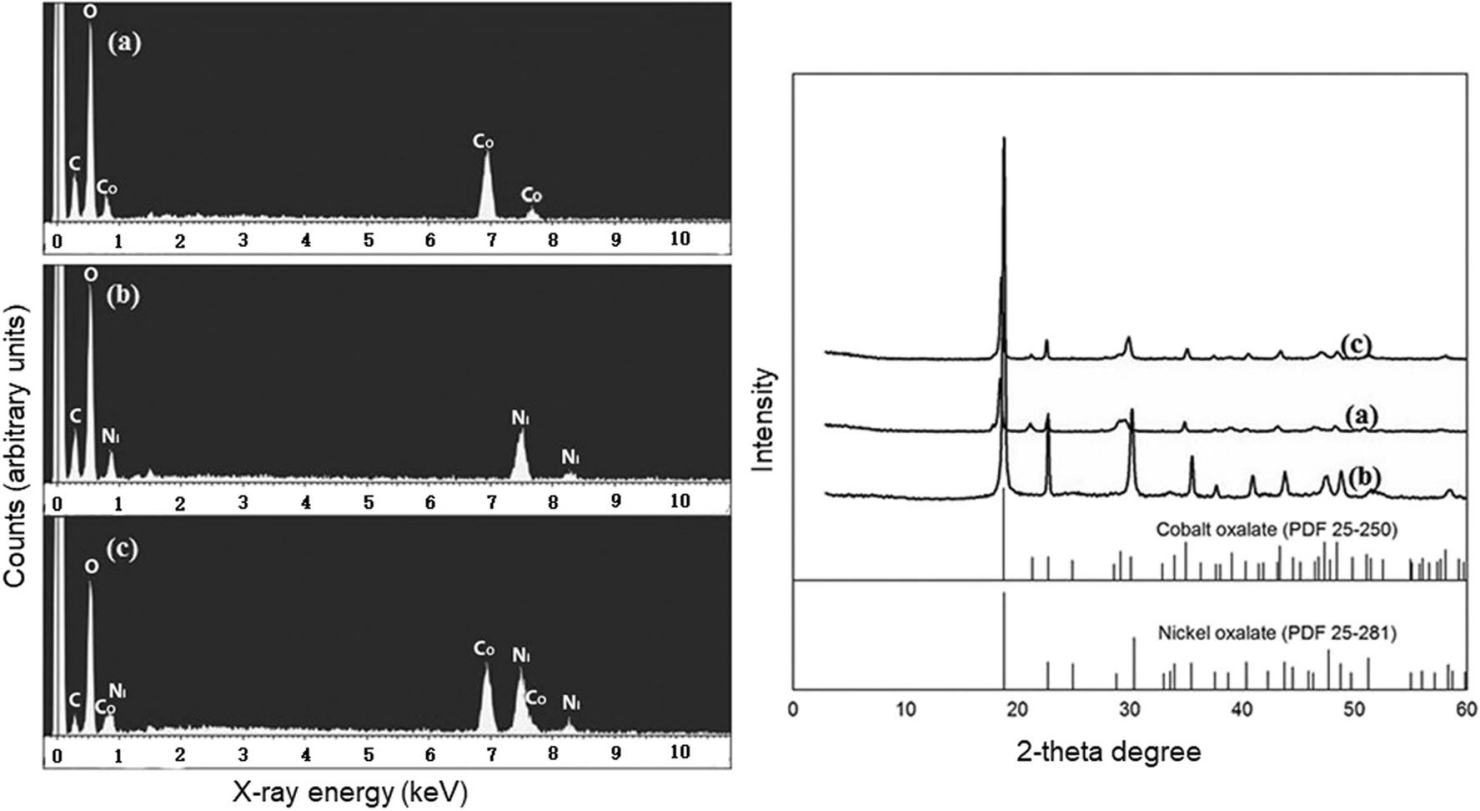
Energy-dispersive X-ray analysis (EDXA) and X-ray diffraction (XRD) of the bioprecipitates using obtained culture supernatants reacted with 10 mM cobalt (**a**), 10 mM nickel (**b**), and mixture of 5 mM cobalt and 5 mM nickel (**c**). Typical images are shown from several separate examinations

Figure 5 shows the morphology of cobalt and nickel crystal precipitates obtained under different experimental conditions. Micro-rod shaped crystals were obtained for cobalt with biomass-free supernatants, EPS solution containing oxalate, and chemically prepared oxalate solution. Micropolyhedrons were observed for nickel when reacted with culture supernatants, EPS solution containing oxalate, and chemically prepared oxalate solution. No obvious differences were apparent between the three treatments for cobalt and nickel precipitation from single metal solutions suggesting that neither supernatants nor the EPS solution had an influence on precipitate morphology in the single metal solutions. However, crystalline morphology observed in the metal mixture was as a smooth layered form using supernatants and EPS solution containing oxalate in contrast to the precipitate from a mixture of nickel and cobalt using chemically prepared oxalate which displayed a rough and non-uniform shape. This demonstrated that the supernatants containing EPS influenced the morphology of precipitated minerals in a mixture of cobalt and nickel compared with chemical oxalate. Figure 6 shows EDXA and XRD of the precipitation obtained using supernatants with single solutions of cobalt, nickel, and their mixture. The elemental composition of C, O, and Co/Ni from EDXA and the XRD pattern of the mineral precipitate using supernatants with single cobalt or nickel solution showed that cobalt or nickel oxalate formed (Fig. 6a, b). For the precipitate produced from the mixture of cobalt and nickel, EDXA showed that it was composed of cobalt, nickel, carbon and oxygen. However, the XRD pattern of a mixed Co/Ni solution showed more similarity with cobalt oxalate (Fig. 6c). This could suggest a difficulty in distinguishing Co and Ni oxalate when precipitating together. In addition, it is possible that a mixed Co-Ni oxalate phase may exist as well as adsorption or other kinds of association with the crystals in poorly or non-crystallized forms.

### Fluorescence properties of EPS from *A. niger*

**Fig. 7:**
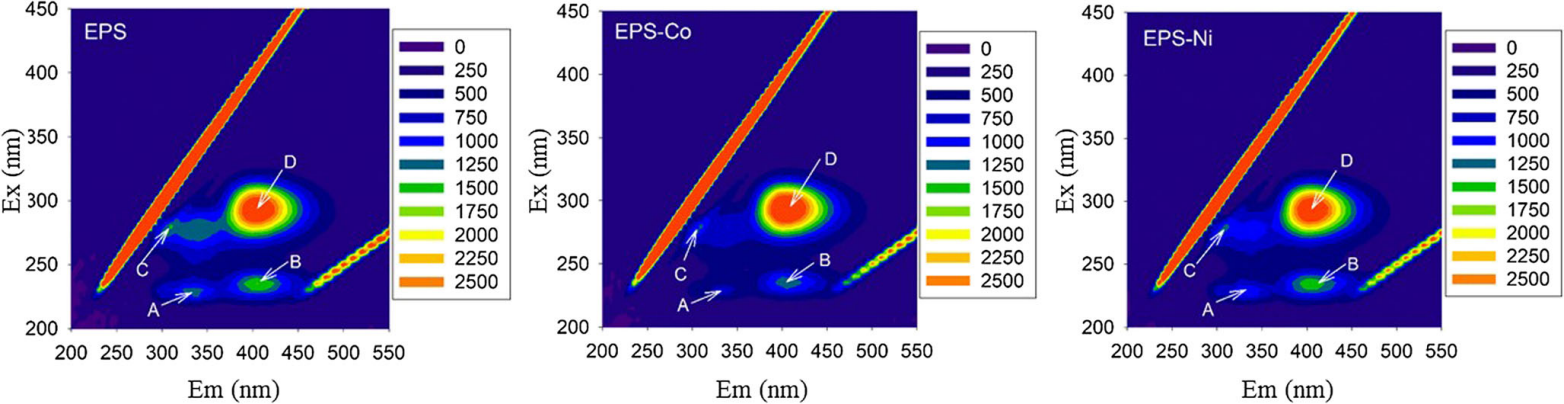
EEM spectra of EPS at 25 °C in the absence and presence of metal ions (5 mM cobalt and nickel). Typical spectra are shown from several separate determinations

Three-dimensional fluorescence spectra of EPS from *Aspergillus niger* in the absence and presence of the metals are shown in Fig. 7. Four fluorescence peaks were obtained in the EEM spectra of EPS. Peaks A (Ex/Em 230/325–340) and B (Ex/Em 235/400–405) can be attributed to aromatic protein-like and hydrophobic acid-like fluorophores, respectively; peak C (Ex/Em 275–280/340) and D (Ex/Em 290–295/405) is attributed to humic acid-like fluorophores (Chen et al., 2003). The fluorescence position and intensity of EPS in the absence and presence of different metal ions at the concentration of 3.67 mM are shown in Table 1. For most fluorescence peaks, the fluorescence intensity decreased after the addition of cobalt and nickel indicating that cobalt and nickel could quench the fluorescence of most fluorescent substances, especially aromatic protein-like (peak A) and humic acid-like (peak C) substances. The fluorescence intensity of hydrophobic acid-like substances (peak B) showed little change after addition of nickel, showing that nickel did not quench its fluorescence but cobalt did. Otherwise, nickel could quench the fluorescence of the other kind of humic acid-like substances (peak D) and the increasing intensity after addition of cobalt indicated that more fluorophores might be unfolded under cobalt stress.

**Table 1:** The fluorescence position (Ex/Em, nm) and intensity (arbitrary units) of EPS in the absence and presence of cobalt and nickel at concentrations of 3.67 mM

System	Peak A		Peak B		Peak C		Peak D	
	Ex/Em	Intensity	Ex/Em	Intensity	Ex/Em	Intensity	Ex/Em	Intensity
EPS	230/335	1070	235/405	1473	275/340	1216	295/405	3198
EPS-Co	230/335	748	235/405	1300	275/340	891	295/405	3353
EPS-Ni	230/340	953	235/400	1435	275/340	888	295/405	3062

### Fluorescence quenching

The fluorescence quenching process can be dynamic quenching, which is due to collision between the fluorophore and quencher, or static quenching due to complexation between the fluorophore and the quencher. The Stern–Volmer Eq. (1) was used to judge whether the quenching processes were dynamic or static (Keizer 1983):

1 $$ {F}_0/F=1+{k}_q{\tau}_0\left[Q\right]=1+{K}_{sv}\left[Q\right] $$

where *F*_0_ and *F* represent the fluorescence intensity in the absence and presence of the quencher, respectively; *K*_q_ is the quenching rate constant; *K*_sv_ is the quenching constant; *τ*_0_ is the average lifetime of the fluorescence in the absence of quencher, which is taken as 10^−8^ s; and [*Q*] is the metal concentration. The values of the quenching constants (*K*_sv_) and *R*^2^ are summarized in Table 2.

**Table 2:** Stern–Volmer fluorescence quenching constant, *K*_sv_, and quenching rate constant, *K*_q_, of EPS in the presence of different metals (cobalt concentration range 0–5.67 mM; nickel concentration range 0–3.67 mM; *R*^2^: determination coefficients)

Quencher		Quenching constants, *K*_sv_ (× 10^3^/M)	Quenching rate constants, *K*_q_ (× 10^11^/M/s)	*R*^2^
Co	Peak A	4.906	4.906	0.9703
	Peak B	1.801	1.801	0.9188
	Peak C	3.850	3.850	0.9772
	Peak D	–	–	–
Ni	Peak A	0.786	0.786	0.2010
	Peak B	–	–	–
	Peak C	2.927	2.927	0.8817
	Peak D	0.592	0.592	0.2531

The quenching rate constant (*K*_q_) values were one order of magnitude larger than the maximum diffusion collision quenching rate constant (2.0 × 10^10^/M/s), indicating that the fluorescence quenching process was mainly governed by static quenching by formation of complexes. However, if the *K*_q_ value was smaller than 2.0 × 10^10^/M/s, this indicated the fluorescence quenching process was dominated by dynamic quenching by intermolecular collisions. According to the quenching constant rates shown in Table 2, it was observed that the fluorescence quenching of EPS by cobalt was due to complexation. The fluorescence quenching of EPS by Ni was both a dynamic (peak A and peak D) and static process (peak C). Furthermore, the quenching strength of cobalt to fluorescence of EPS was larger than that for nickel.

### Binding constants and binding sites

For ligand molecules that bind independently to a set of equivalent sites on a macromolecule, the equilibrium between free and bound molecules is given by the Hill (2013) Eq. (2):

2 $$ \mathit{\log}\left[\left({F}_0-F\right)/F\right]=\mathit{\log}{K}_b+ nlog\left[Q\right] $$

where *F*_0_ and *F* are the fluorescence intensities in the absence and presence of quencher, respectively; *K*_b_ is the binding constant; *n* is the number of binding sites; and [*Q*] is the concentration of metal. The binding constant (*K*_b_) reflects the interactive intensity between EPS and the metal.

The binding constants (log *K*_b_) and the number of binding sites (*n*) of the EPS-metal system are listed in Table 3. The aromatic protein-like (peak A) and hydrophobic acid-like substances (peak B) possessed cobalt binding ability but exhibited no complexation with nickel. Both the binding ability and binding sites of cobalt with humic acid-like substances (peak C) were much larger than those for nickel. The maximum value of log *K*_b_ was obtained from the EPS-cobalt system at peak B, indicating that hydrophobic acid-like fluorophores possessed a strong binding ability for cobalt.

**Table 3:** Binding constants (log*K*_b_) and binding sites (*n*) of peaks A, B, and C from EPS complexation with different metals (cobalt concentration range 0–5.67 mM; nickel concentration range 0–3.67 mM; *R*^2^: determination coefficients)

Metal ion		Binding constant, log *K*_b_	Binding sites *n*	*R*^2^
Co	Peak A	3.56	1.62	0.9564
	Peak B	6.65	3.16	0.9664
	Peak C	3.20	1.51	0.9575
Ni	Peak C	1.51	0.85	0.8517

## Discussion

Bioprecipitation is regarded as a potential technique for the removal and recovery of metals from solution (Gadd et al. 2014; Gadd and Pan 2016; Liang and Gadd 2017; Torres-Aravena et al. 2018). For example, carbonates, oxides, phosphates, and oxalates produced by microbial activities can transform metals from soluble species into the corresponding insoluble compounds via precipitation (Li and Gadd 2017a, b; Liang and Gadd 2017; Peng et al. 2018). Oxalic acid is produced by a wide variety of fungi, and this plays an important role in metal detoxification, weathering and cycling of metals, bioleaching, and biomineralization (Anjum et al. 2010; Fomina et al. 2008; Gadd 2007; Gadd et al. 2014; Kang et al. 2019; Nancharaiah et al. 2016; Yang et al. 2019). Metal oxalate crystal formation has been widely found using *Aspergillus* species, such as zinc oxalate (Sutjaritvorakul et al. 2016), lanthanum oxalate (Kang et al. 2019), manganese oxalate (Wei et al. 2012), and calcium oxalate (Pinzari et al. 2010). Chemically produced oxalate has also been used as a precipitant for recovery of valuable metals from a bioleaching solution of spent lithium-ion batteries (Sun and Qiu 2012) and as a leaching solution for monazite (de Vasconcellos et al. 2006). Copper recovery can reach 99.5% using chemical oxalate precipitation (Gyliene and Salkauskas 1995). Cobalt recovery from a spent lithium-ion batteries leachate could reach ~ 93% using chemical oxalate (Chen et al. 2011). In this work, approximately 94% and 98% cobalt recovery from a 10-mM solution were obtained using biomass-free *A. niger* culture supernatants and chemical oxalate, respectively, thus demonstrating that supernatants from *A. niger* were almost as efficient as chemical means to recover cobalt via bioprecipitation. Moreover, culture supernatants were more efficient for recovery of nickel compared with the chemical oxalate treatment. In a Co/Ni mixture, cobalt and nickel recoveries were increased compared to the single metal treatments. However, the apparent dominance of cobalt oxalate in the mixed Co/Ni precipitate could be explained by the solubility product constants (*Ksp*) of the two oxalate minerals. The *Ksp* of cobalt oxalate (2.70 × 10^−9^) is significantly smaller than that of nickel oxalate (1.2 × 10^−3^) (IUPAC-NIST Solubility Database).

Extracellular polymeric substances (proteins and/or polysaccharides) can play an important role in biomineralization, regulating and controlling nucleation, and growth of crystal structures (Ercole et al. 2012; Kawaguchi and Decho 2002; Li and Gadd 2017a, b; Perri et al. 2018; Tourney and Ngwenya 2014). In our study, it was found that the supernatants, or EPS obtained from supernatants, did not exert a significant influence on crystal morphology compared with chemical methods during the formation of cobalt and nickel oxalate. Only for the Co/Ni–mixed solution was there a difference in crystal morphology between those derived from the supernatants and the chemical system (Fig. 5). In other studies, fungal growth supernatants from *Neurospora crassa* were found to greatly influence the scale of crystal morphology for metal carbonates (Li and Gadd 2017a, b). Although EPS did not change the morphology of the nickel precipitate in the single metal solution, it did influence nickel recovery (Fig. 3).

In other studies, significant amounts of extracellular protein in fungal supernatants were removed by precipitation of copper carbonate (Li and Gadd 2017a, b; Liu et al. 2019). Here, extracellular protein was only partly removed during oxalate precipitation, while the extracellular polysaccharide concentration did not show much change compared to the control. Much more protein was removed with cobalt compared to nickel. It is well known that extracellular polymeric substances contain a variety of metal-binding groups (Wang et al. 2014; Liu et al. 2015; Song et al. 2016). Lower binding affinities between nickel and EPS could lead to a decrease in recovery of Ni in the presence of EPS in the oxalate solution compared to cobalt (Fig. 3). The aromatic protein-like, hydrophobic acid-like substances and humic acid-like substances (peak C) had a clear binding affinity for cobalt. Aromatic protein-like and humic acid-like substances have been found to easily trap copper (Wang et al. 2015; Wei et al. 2017). The differences in affinities of these substances for metals could explain the differences in the recovery efficiency for cobalt and nickel influenced by EPS. This work has demonstrated that fungal derived oxalate can be used for the recovery of cobalt from solution and provides insights into the role of other extracellular products during this process.
